# Vocal Emotion of Humanoid Robots: A Study from Brain Mechanism

**DOI:** 10.1155/2014/216341

**Published:** 2014-01-22

**Authors:** Youhui Wang, Xiaohua Hu, Weihui Dai, Jie Zhou, Taitzong Kuo

**Affiliations:** ^1^Department of English, Tamkang University, New Taipei City 25137, Taiwan; ^2^The First Affiliated Hospital, Second Military Medical University, Shanghai 200433, China; ^3^School of Management, Fudan University, No. 220 Handan Road, Shanghai 200433, China

## Abstract

Driven by rapid ongoing advances in humanoid robot, increasing attention has been shifted into the issue of emotion intelligence of AI robots to facilitate the communication between man-machines and human beings, especially for the vocal emotion in interactive system of future humanoid robots. This paper explored the brain mechanism of vocal emotion by studying previous researches and developed an experiment to observe the brain response by fMRI, to analyze vocal emotion of human beings. Findings in this paper provided a new approach to design and evaluate the vocal emotion of humanoid robots based on brain mechanism of human beings.

## 1. Introduction

People have long been dreaming of building brain-like intelligent machines. Ever since the first Artificial Intelligence conference taking place in 1956, AI development has been over half a century old. While the coming-ups of Kismet [1] and Cog [2, 3] by MIT have served as hallmarks of AI technology in the field of humanoid robot design, in China, a project to design a robot with sophisticated interactive system was granted by the government. Although previous papers contribute significant efforts into humanoid robot design, the majority of them focus on facial expression or recognition [4].

With the increasingly strict demands of robots, it is suggested that more attention should be shifted into the issue of vocal emotion design of AI robots to facilitate the communication between man-machines and human beings. To upgrade the voice design of robots, it is necessary to have a look at the mechanism for processing vocal emotions of human beings and the acoustic features of vocal stimuli. With this, it is expected that, apart from facial expression and recognition, future designs for interactive robots are not only emotionally rich in vocal expression but also able to perform vocal emotion recognition.

## 2. The Mechanism of Vocal Emotion

### 2.1. Prosody Comprehension

The mechanism of prosody comprehension is proposed to be mediated by a sequential multistep process unfolded from basics stages of acoustic voice analysis (bound to temporal brain areas) and proceeding to higher-level stages of categorization and recognition (associated with frontal aspects of the brain). Following the processing of auditory information within the ear, brainstem, thalamus, and primary acoustic cortex (A1), three successive steps of prosody decoding can be identified:

 Step 1: extraction of acoustic features of prosodic cues;
 Step 2: identification of vocally expressed emotion by means of multimodal integration;
 Step 3: explicit evaluation and cognitive elaboration of vocally expressed emotions.

Each of these steps, in turn, is differentially represented in the human brain: whereas the extraction of acoustic features has been linked to voice-sensitive structures of the middle part of superior temporal cortex (m-STC), more posterior aspects of the right STC have been recognized for their contribution to the identification and integration of emotional signals into a common percept [5]. Beyond anatomical characterizations, research has expanded its focus to include the functional role of *temporal voice areas *in a variety of voice cognition skills (i.e., abilities to extract, evaluate, and categorize nonlinguistic information available in voices) [6]. Particularly the ability to decode vocal emotional information, or more precisely the contribution of temporal voice areas to the process of inferring emotions from vocal cues, constitutes a field of research that has attracted attention over the past years.

The ability to correctly interpret emotional signals from others is crucial for successful social interaction. A wealth of neuroimaging studies have indicated that voice sensitive auditory areas [6–8] activate a broad spectrum of vocally expressed emotions more than neural speech melody (prosody). Previous fMRI studies relying on standard data analyses have indicated that the middle part of the superior temporal gyrus (STG) reacts more strongly to various vocal emotions [9–12] than to neutral prosody. Recently, voice-sensitive responses of the temporal lobe have become a subject of thorough scientific scrutiny, and findings suggesting a unique role of the superior temporal cortex (STC) in human voice perception have been replicated multiple times. Research has established a contribution of brain structures including the p-STC and m-STC and the DLPFC and OFC as well as limbic regions such as the amygdale or aspects, of the arMFC. Each of these brain structures, in turn, has been suggested to be associated with distinct aspects of prosody decoding from basic stages of acoustic analysis to higher-order evaluative processes as Table 1 [5].

In this table, *T*-values and MNI coordinates (in square brackets) of highest activated voxels within each region are presented: SMA, supplementary motor cortex; ACC, anterior cingulate cortex; DLFC, dorsolateral frontal cortex; OBFC, orbitobasal frontal cortex; IPL, inferior parietalis lobulus; STG, superior temporal gyrus; MTG, middle temporal gyrus. Respective Brodmann Areas (BA) are printed in brackets; by SPM99, random-effect analysis, *n* = 10, P b 0.05 corrected, T N 4.30, k N 38.

According to the neuroanatomical model proposed by Ross [13], prosodic information is processed within distinct right-sided perisylvian regions that are organized in complete analogy to left-sided language areas. Furthermore, a closer look at the current literature available on the topic, however, underpins that the contribution of temporal voice areas represents just one piece of the puzzle of how the human brain recognizes and comprehends vocal emotional information. In fact, the processing of vocal expressions of emotions (e.g., speech prosody) appears to depend on a network incorporating not only voice-sensitive areas, but also posterior temporal, frontal, and subcortical brain structures.

Among previous research on vocal emotion, one has delineated the cerebral network engaged in the perception of emotional tone, with functional magnetic resonance imaging (fMRI) performed during recognition of prosodic expressions of five different basic emotions (happy, sad, angry, fearful, and disgusted). As compared to baseline at rest, the results indicated widespread bilateral hemodynamic responses within frontal, temporal, and parietal areas, the thalamus, and the cerebellum. A comparison of the respective activation maps, however, revealed comprehension of affective prosody to be bound to a distinct right-hemisphere pattern of activation, encompassing posterior superior temporal sulcus (Brodmann Area (BA) 22), dorsolateral (BA 44/45), and orbitobasal (BA 47) frontal areas. These findings indicate that part of distinct cerebral networks subserve processing of intentional information during speech perception [14]. Taken together research results presented define prosody comprehension as a complex function tied to several cortical and subcortical brain structures.

Findings of research [14] suggested that the correct responses of each valence varied. “Happy,” “Angry,” and “Sad,” seemed to be recognized easily, while the valences “fearful” and “disgusted” appeared to be more difficult for people to recognize correctly. The reasons for these results still require more empirical studies. Nevertheless, for a more satisfactory recognition rate, while designing vocal emotion interactive systems for future humanoid robots, the complicity of individual emotion shall be taken into consideration. The percentage of correct answers during identification of emotional was intonation (mean: 75.2 F 7.9%). Recognition rates for specific emotions ranged between 51% (fear) and 92% (happiness) [14].

### 2.2. Vocal Emotion by fMRI Studies

In recent years, there has been an explosion of research into the neural mechanism of human beings' emotions. The brain regions regarding to vocal emotions can be identified with the functional neuroimaging techniques such as fMRI (functional Magnetic Resonance Imaging) that measure hemodynamic changes. Previous fMRI studies relying on standard data analyses have indicated that the middle part of the superior temporal gyrus (STG) reacts more strongly to various vocal emotions [9–12] than to neutral prosody. Recently, voice-sensitive responses of the temporal lobe have become a subject of thorough scientific scrutiny, and findings suggesting a unique role of the superior temporal cortex (STC) in human voice perception have been replicated multiple times. Research has established a contribution of brain structures including the p-STC and m-STC and the DLPFC and OFC as well as limbic regions such as the amygdale or aspects, of the arMFC. Each of these brain structures, in turn, has been suggested to be associated with distinct aspects of prosody decoding from basic stages of acoustic analysis to higher-order evaluative processes [5].

According to the neuroanatomical model proposed by Ross [13], prosodic information is processed within distinct right-sided perisylvian regions that are organized in complete analogy to left-sided language areas. Furthermore, a closer look at the current literature available on the topic, however, underpins that the contribution of temporal voice areas represents just one piece of the puzzle of how the human brain recognizes and comprehends vocal emotional information. In fact, the processing of vocal expressions of emotions (e.g., speech prosody) appears to depend on a network incorporating not only voice-sensitive areas, but also posterior temporal, frontal, and subcortical brain structures.

Among previous research on vocal emotion, one has delineated the cerebral network engaged in the perception of emotional tone, with fMRI performed during recognition of prosodic expressions of five different basic emotions (happy, sad, angry, fearful, and disgusted). As compared to baseline at *r*-test, the results indicated widespread bilateral hemodynamic responses within frontal, temporal, and parietal areas, the thalamus, and the cerebellum. A comparison of the respective activation maps, however, revealed comprehension of affective prosody to be bound to a distinct right-hemisphere pattern of activation, encompassing posterior superior temporal sulcus (Brodmann Area (BA) 22), dorsolateral (BA 44/45), and orbitobasal (BA 47) frontal areas. These findings indicate that part of distinct cerebral networks subserve processing of intentional information during speech perception [14].

### 2.3. Acoustic Features of Vocal Stimuli

During speech production, information about a speaker's emotional state is predominantly conveyed by the modulation of intonation (affective prosody). At the perceptual level, emotional tone is characterized by variations of pitch, syllable durations, loudness, and voice quality across utterances (suprasegmental features) imposed upon segmental verbal information encoded in phonetic/phonological units [15–19]. Among the mounting acoustic features that have been studied, primarily four parameters have emerged as prime candidates to subserve the vocal signaling of emotions [6, 20, 21].

1. Voice intensity corresponding to the perceived loudness of a given vocal signal.
2. Tempo and pausing corresponding to the rate of vocalization (e.g., speech rate or laughter rate).
3. Fundamental frequency of vocal fold vibration (F0) defining the perceived pitch of a voice.
4. Energy distribution in the frequency spectrum (i.e., relative amount of energy within predefined frequency bands affecting voice quality) [22].

Rigorous analysis of acoustic cues measured from samples of emotional speech helped to define distinctive acoustic profiles for a set of central emotions such as anger, joy, fear, or sadness. The corresponding findings are described in the literature as the following [6, 17, 20, 21, 23, 24]: as *anger *generally has been described to be indexed by increasing voice pitch accompanied with increases in loudness; *sadness*, for instance, has been characterized to show decreases in voice pitch, speech rate, and loudness of a speakers voice. *Fear*, on the other hand, can be revealed by increases in voice pitch combined with increases in speech rate, while *joy *has been related to increases in voice pitch, loudness, and speech rate. Similar results have been obtained for various nonverbal vocalizations, which have been associated with distinct acoustic profiles which demonstrate the sophisticated interplay of acoustic properties related to voice quality, pitch, and intensity [25, 26].

In the design of vocal emotions of a humanoid robot, there are two problems involved: (1) the expression of speech emotion, this can be solved by choosing certain emotional words and adopting proper speech synthesis technology; there are a lot of research achievements in this field; (2) the recognition of human beings' emotions, this will help the humanoid robot to make appropriate reactions in interactive process; it needs to process the human beings' speech signals through emotional pattern recognition methods.

For a humanoid robot, the acoustic features of received speech signals should be described by some extracted characteristic parameters. On this respect, Linear Prediction Cepstrum Coefficient (LPCC) and Mel Frequency Cepstrum Coefficient (MFCC) have been widely used as parametrically representing speech signal for speech recognition [27]. LPCC computing method is a recursion from LPC Parameter to LPC cepstrum according to All-pole model. However, The LPCC parameter cannot adequately reflect the human auditory characteristics. It inherits defects of LPC, that is, all frequencies based on composite coefficients tend to be linear and including noise details at high frequencies segment. In fact, the human auditory system is a special nonlinear system so as to respond to the different frequency signals. MFCC is based on the known variation of the human ear's bandwidths, it spaced linearly below 1000 Hz frequencies and logarithmically above 1000 Hz frequencies, and the mechanism of input speech signal matched with auditory characteristics. Experiences show that the performance of MFCC parameter is better than that of LPCC parameter [27].

In order to identify vocal emotions of human beings, it is necessary for us to adopt appropriate pattern recognition methods on the processing of speech signals. The state-of-the-art in pattern recognition techniques includes Dynamic Time Warping (DTW), Hidden Markov Modeling (HMM), and artificial neural networks (ANN). Dynamic Time Warping (DTW) is an early and classical algorithm which based on the thought of dynamic optimization and can resolve the matching problem of the difference of speech's length. In the isolated word speech recognition, the disadvantage of HMM and ANN is limitation of heavy computations and training templates that are required for higher accuracy, but the DTW algorithm training hardly needs the additional calculations. So DTW approach is usually used in the recognition of speech signals due to ease of implement and flexibility [27]. In this regard, the brain's emotional mechanism has provided the important basis for the design of pattern recognition.

## 3. Experiment Observations

### 3.1. Experiment Model

**Figure 1:**
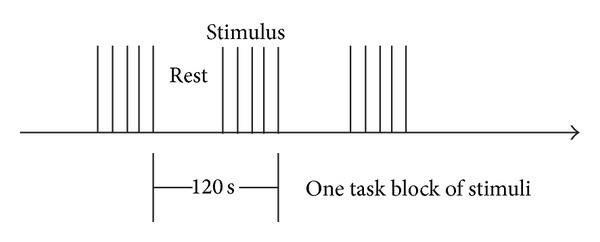
One task block duration of each stimulus event.

To have a better look at mechanism for processing vocal emotions of human beings, we designed an experiment to demonstrate appropriate picture and text stimuli in use of f-MRI method. As seen from Figure 1, multiple task blocks will be designed in certain cycle; each task consists of one or more pairs of rest and stimulus state. In the rest state, subjects will not accept any stimuli, and in the stimulus condition, subjects will receive the same and approximate continuous stimulus events. In this experiment, 3 kinds of stimuli blocks including text stimuli block, picture materials block, and graphic text stimuli block were selected (three runs).

In order to generate appropriate verbal stimuli, 16 listening questions (2  runs) adopted from Part 1 of China Accreditation Test for Translators and Interpreters 2 (Catti 2) with emotionally neutral content were selected; all were news read by professional broadcasters. Materials were tested in a pilot study by 52 college students in Fudan University, China, to achieve appropriate level of difficulty. The subjects were required to pass a test split from the stimuli test, to confirm their ability to understand the question. Duration of each verbal recording ranged from 35 s to 40 s.

Tape recordings of all 16 utterances were presented to 6 healthy subjects (2 males, 4 females, aged 22–28, all right-handed) without history of neurological or psychiatric diseases participated in the fMRI experiment and comparison of brain conditions were recorded as they process the stimuli.

They lay supine in a 3-T whole body scanner (Siemens Vision), their eyes opened and their heads supported by a foam rubber within the head coil. Each session in the design included the part of rest for 30 seconds, the duration of each block lasted 120 s.

Subjects were instructed to listen to the contents and to answer the following True/False questions. The results of the answering were not calculated for the reason that part of compulsory answering was suggested by previous research as a method to help subjects to focus while listening to the stimuli. They lay supine in a 3-T whole body scanner (Siemens Vision), their eyes closed and their heads supported by a foam rubber within the head coil, button placed under their index and middle fingers for them to choose the answers.

**Figure 2:**
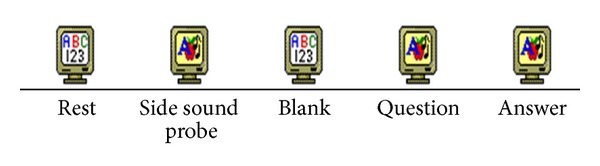
Experiment steps designed by E-prime2.0.

Each session in the design included the part of rest for 24 seconds, stimuli for 35–40 seconds, blank for 2 seconds, question reading part for 10 seconds, and answering 2 seconds, two runs in total, lasting for 540 seconds, designed by E-prime2.0 as Figure 2.

### 3.2. Experiment Findings

**Figure 3:**
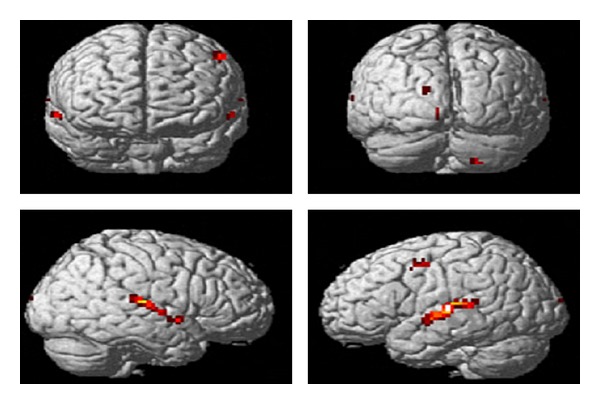
Listening question identification.

Findings of the present research was in line with the comparison revealed in previous research, indicating that the right auditory cortex is more sensitive to tonality, while the left auditory cortex has been shown to be more sensitive to minute sequential differences in sound, such as in speech. Comparing findings of the present study and those of [14], we can notice that while the materials of the former are highly speech-like, the left BA 41 was activated significantly. On the contrary, the stimuli of the latter was tended to be more tonal like (affective prosody), resulting great activation in right BA 41. It is thus assumed that, although STG are generally recognized to be related to auditory function in human brain, lateralization of activation in this area still depended on the categories of the input signals. As in Figure 3 (SPM99, random-effect analysis, T N 4.30, k N 38, P b 0.05, corrected), significant hemodynamic responses during identification of sentence listening. The listening task yielded specific activation within the left superior temporal gyrus (STG, BA 41, *Z* = 5.5).

**Figure 4:**
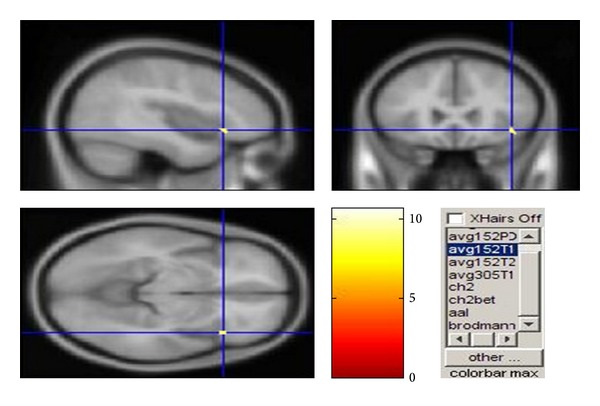
Plane graph of activity region inside brain.

We notice that when some displeasure voice was heard, the task yielded specific activation within the brain Inferior Frontal Gyrus area (shown in Figure 4, center coordinates: 42, 24, and −9), indicating that the subjects were in the high attention and caused a certain amount of dissatisfaction affection. Based on the experimental results, we can get typical human brain activation pattern and activation level under different situational context of vocal information stimulations, from which to obtain statistical features and the related empirical data of people's emotion.

For future design of voice system for humanoid robot, it is suggested that, depending on functions of individual robots, the voice shall be adjusted, according not only to acoustic features of voice (voice intensity, loudness, tempo, pausing, corresponding to the rate of vocalization, fundamental frequency of vocal fold vibration (F0), pitch, frequency spectrum, etc.), but also to their tendency toward tonality or speech. For example, home robots shall put more emphasis on their prosody decoding system, since the context fit the application tend to be soft and informal, while vocal system design for robots in public areas (school, museums, tourist attractions, etc.) shall be with emphasis on speech content rather than vocal emotion. The former tends to be more right-hemisphere oriented and the latter tends to be more left-hemisphere oriented. With this regard, the comparison between the findings of the present research and those of [14] may serve as a valuable reference.

We have already applied these findings to the infants' speech recognition [27]. Infants are not able to use the languages that adults can understand to express their physiological and psychological states, so their expressions, gestures, actions, and speeches have been not only the methods to communicate with the outside world, but also the important information sources of reflecting their emotions and needs, which imply health status and mental development level. As for the basic emotions and needs caused by human physiological factors, whether infants or adults, their brain's response characteristics are consistent by fMRI observations. This will help us better understand the infants' emotion and needs and therefore develop the effective method of pattern recognition through their speech signals.

We selected 3 kinds of speech signals that reflect normal emotions and needs of infants such as happy, hungry, and sleepy which are very typical as well as easy for evaluation and acquisition in infants' daily life. The daily activity information of infants (such as the time for drinking, excreting, etc.) and the environment information (such as the temperature, voice, etc.) were also recorded to assist the recognition; test results showed that the average recognition rate may be achieved more than 80%. This technology can be applied to the design of baby care humanoid robot with emotional intelligence.

## 4. Conclusions

While contemporary technology of humanoid robot design have contributed greatly to the topic of emotion recognition as well as AI, only marginal attention has been paid to the vocal domain. Exploring the previous fMRI research on vocal emotion recognition has deepened our understanding about how the mechanism of vocal emotion processing works in the human brain, such as the contribution of right-sided temporal and frontal regions to the processing of emotional prosody independent of specific emotional categories. It will provide the inspiration to the design of humanoid robot with emotional intelligence, which has wide potential application such as emotion analysis in emergency events [28] and all kinds of smart services [29].

## Figures and Tables

**Table 1:** Hemodynamics activation during identification of emotions and vowels.

		Emotions versus baseline	Vowels versus baseline	Emotions versus vowels	Vowels versus emotions
SMA/ACC (BA 6, 24, 32)		9.28 [3, 9, 57]	11.97 [3, 9, 51]	—	—
DLFC (BA 9, 44–46)	Left	9.52 [−48, 9, 30]	15, 14 [−54, 6, 30]	—	7.18 [−54, 6, 33]
	Right	16.89 [51, 33, 15]	12.42 [42, 0, 33]	—	—
OBFC (BA 47)	Left	7.67 [−45, 30, −12]	—	—	—
	Right	7.33 [48, 36, −18]	—	8.63 [48, 33, −15]	—
Rolandic area (BA 3/4/6)	Left	12.08 [−45, −9, 18]	10.11 [−57, 0, 21]	—	—
	Right	13.29 [45, 3, 24]	10.16 [57, 3, 18]	—	10.16 [−48, −39, 54]
IPL (BA 7, 40)	Left	—	14.15 [−21, −63, 48]	—	10.57 [27, −66, 39]
	Right	9.89 [39, −51, 48]	15.99 [27, −54, 57]	—	—
STG (BA 22, 41, 42)	Left	8.96 [−60, −45, 9]	8.10 [−60, −30, 9]	—	—
	Right	12.32 [48, −45, 6]	8.18 [51, −42, 6]	8.69 [48, −42, 3]	—
MTG (BA 21)	Left	11.18 [−36, −3, −15]	8.17 [−39, −9, −18]	—	—
	Right	16.18 [45, 15, −30]	11.68 [54, 3, −15]	—	—
Thalamus	Left	11.44 [−6, −27, −3]	12.13 [−6, −24, −3]	—	—
	Right	15.75 [6, −21, 0]	12.89 [9, −21, 6]	—	—
Cerebellum	Left	13.56 [−30, −60, −27]	7.98 [−33, −54, −33]	—	—
	Right	10.86 [42, −63, −33]	9.18 [48, −66, −33]	—	—
	Vermis	14.73 [−6, −72, −30]	10.42 [−3, −72, −24]	—	—
